# Incidental finding of thymic cyst in a newborn baby

**DOI:** 10.1002/ccr3.2905

**Published:** 2020-04-28

**Authors:** Elmas Shaqiri, Gentian Vyshka

**Affiliations:** ^1^ Department of Forensic Pathology Institute of Legal Medicine Tirana Albania; ^2^ Biomedical and Experimental Department Faculty of Medicine University of Medicine in Tirana Tirana Albania

**Keywords:** cysts, mediastinal formation, newborn, thymus hyperplasia

## Abstract

Thymic hyperplasia is a common finding among newborns, but cyst formation might produce mediastinal symptoms. Lethal outcome is reported when accompanied by massive intrathoracic hemorrhage. The clinical picture in our case was characterized from profuse multiple hemorrhagic foci and respiratory distress syndrome, with an unclear role of the thymic cyst.

## Graphical abstract

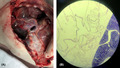

## 1 INTRODUCTION

The images are related to the autopsy findings of a male child, born alive but who died approximately fifteen minutes after the vaginal delivery. The pregnancy was at its term and the mother had two other healthy children before the third delivery. The child weighted 3300 grams, with cephalic presentation; he breathed spontaneously but some minutes later deep cyanosis and lack of pulse prompted resuscitation efforts. The APGAR score of three was registered a quarter hour after the birth and some moments later he was pronounced dead.

**Figure 1:**
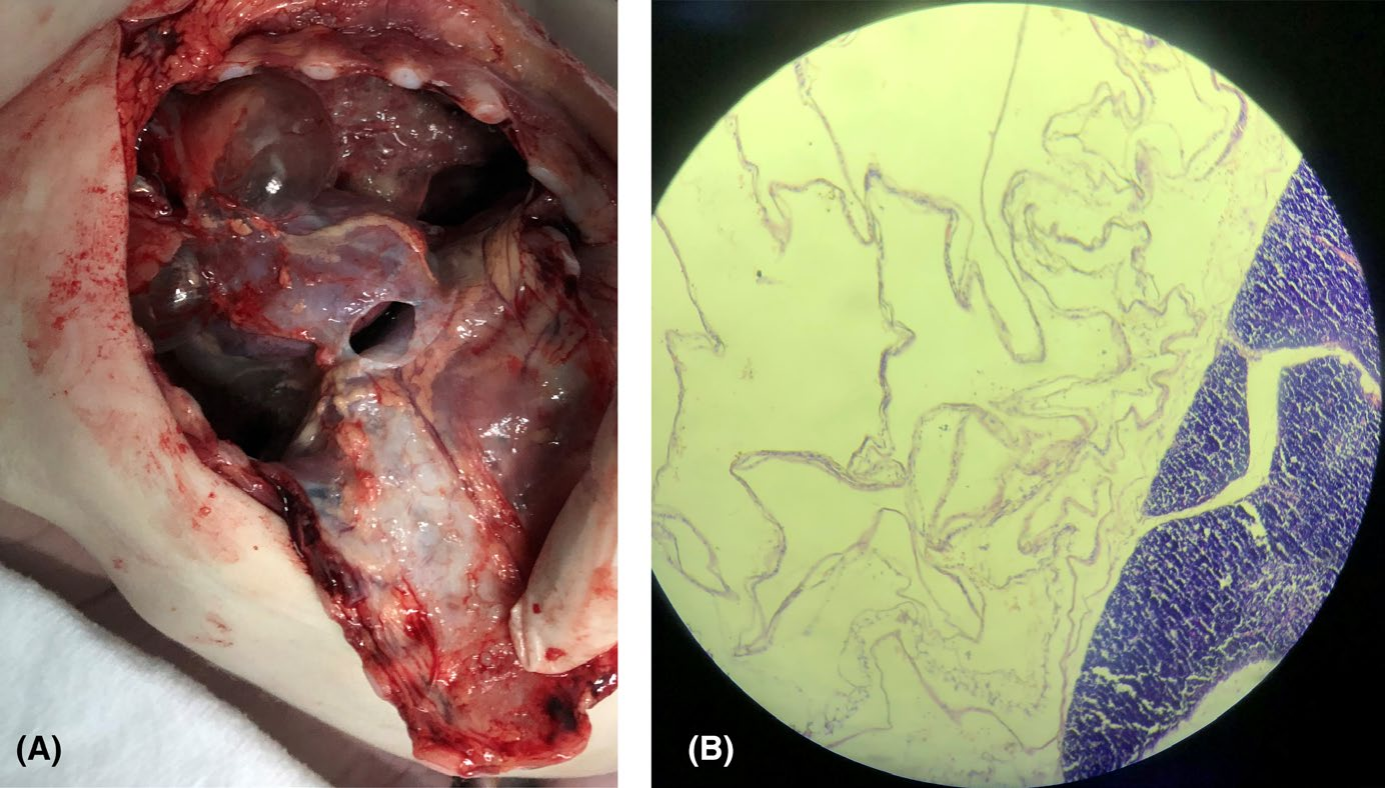
A, a mediastinal formation with a bilocular thymic cyst was seen during the autopsy. B, A macro‐follicular formation inside the thymus with endothelial hyperplasia and coexistence of several micro‐follicular glandular structures [hematoxylin and eosin, ×160]

A thorough autopsy was performed; profuse hemorrhagic foci were found in almost all of his viscera. A retrosternal plum‐like bilocular cystic formation was seen and photographed (Figure 1A). The cystic formation was mainly air‐filled, with some light‐pink liquid leaking after its section. The microscopy of the cyst revealed a follicular structure with eosinophilic fluid inside (Figure 1B). The cyst measured sagitally 3 × 2.6 cm in the left lobe and 2.8 × 2.4 cm in the right lobe, when emptied during autopsy. No direct compressive effects on the respiratory tree could be demonstrated.

There are reports of respiratory distress syndrome among infants related to thymic hyperplasia.^1^ Thymic hyperplasia must be the most common from mediastinal masses diagnosed among newborns. Birth trauma, hemorrhagic disease, coagulopathy, erythroblastosis fetalis are listed among the serious background disorders leading to an unfavorable or even lethal outcome.^2^

## CONFLICT OF INTEREST

None declared.

## AUTHOR CONTRIBUTIONS

ES and GV: involved in manuscript writing, data collection, and literature reviewing.
